# Therapy of Pyothorax in Cats via Small-Bore Thoracostomy Tube in Terms of Efficacy, Complications and Outcomes

**DOI:** 10.3390/ani12010107

**Published:** 2022-01-03

**Authors:** Evelyn Heier, Gabriel Wurtinger, Esther Hassdenteufel, Matthias Schneider

**Affiliations:** Department of Veterinary Clinical Sciences, Small Animal Clinic, Justus-Liebig-University Giessen, 35392 Giessen, Germany; wurtinger@kleintierkardiologie-giessen.de (G.W.); matthias.a.schneider@vetmed.uni-giessen.de (M.S.)

**Keywords:** pyothorax, cat, thoracostomy tube, chest drain, small-bore, closed-chest lavage, unfractionated heparin, alteplase

## Abstract

**Simple Summary:**

With this study we present our therapeutic strategy for cats with purulent fluid accumulation in the thorax. In addition to the systemic administration of antibiotics, the aim of the therapy is always the drainage of the purulent fluid from the thorax. For this purpose, we use a particular small-bore chest drain. The first aim of our study is to assess the efficacy and complication rate of our drainage. The second objective is to evaluate two treatment groups regarding their disease outcomes. We were able to show that our small-bore chest drain is similarly effective to the traditionally used large-bore drains. At the same time, we had a very low drain-associated complication rate. We detected no difference between the treatment groups and, thus, no effect on survival by early placement of bilateral drains into the thoracic cavity or lavage of the thoracic cavity with a heparinised solution. Our study supports the theory that drainage of purulent fluid from the thoracic cavity in cats can be performed with small-bore drains with good results and minimal risk of complications.

**Abstract:**

First-line therapy for cats with pyothorax consists of intravenous antibiotics, drainage of the septic pleural effusion and closed-chest lavage. Large-bore thoracostomy tubes are traditionally used for drainage, but case series indicate a comparable efficacy using small-bore tubes. In this retrospective study, we describe a new technique of sheath-guided small-bore (6 F) thoracostomy tubes in cats with pyothorax and evaluate their efficacy and complications. Additionally, we compare outcomes between two treatment groups. Placement and use of the small-bore thoracostomy tubes described here has a low complication rate of 4% (3/67 tubes), and 53% (24/45) of the cats could be treated with thoracostomy tubes and closed-chest lavage according to the protocol. The success rate is reduced by 18% (8/45) due to deaths caused mainly by sepsis, 16% (7/45) due to structural diseases requiring surgery and a further 14% (6/43) due to lavage failures that could only be cured after additive therapy (thoracotomy or fibrinolysis). The long-term prognosis was very good, with a survival rate one year after discharge of 94% (30/32). We detected no effect on survival by early placement of bilateral thoracostomy tubes or closed-chest lavage with a heparinised solution. In conclusion, therapy of pyothorax with small-bore thoracostomy tubes is as successful as therapy with large- or medium-bore tubes.

## 1. Introduction

Pyothorax is a life-threatening disease. The overall prognosis for cats with pyothorax is fair to good. Cats survive to discharge in 46% to 78% of cases [1,2,3,4,5]. The mortality rate is highest during the first 48 h of hospitalisation [1,2,6].

Treatment modalities include medical management and surgery. A combination of drainage of the septic pleural effusion via thoracostomy tubes and intravenous (IV) administration of a broad-spectrum antibiotic is currently the first-line treatment. Surgical management is recommended in 4% to 6% of cases because of intrathoracic abscesses [1,2,5] detected by diagnostic imaging and in 5% to 9% of cases because medical management fails [1,2,3,4].

For medical management, single or multiple thoracentesis or placement of a thoracostomy tube with and without intermittent closed-chest lavage are described [1,2,3,4]. Traditionally, large-bore thoracostomy tubes (14–16 F) are placed with a rigid trocar under anaesthesia [4,5,7]. Complications associated with large-bore thoracostomy tubes in cats with pyothorax include anaesthetic complications, pneumothorax, lung laceration and subcutaneous fluid leakage [4,5].

In addition to the classical large-bore tubes, the use of a 10 F trocar-based thoracostomy tube placed under general anaesthesia is published [3]. Alternatively, the use of a small-bore (6 F) wire-guided thoracostomy tube is reported in 2 case series of cats with pyothorax [8,9]. The most common complication with these small-bore tubes is a failure to drain, as a result of kinking [8].

The aim of this retrospective study is to describe a new technique of sheath-guided small-bore (6 F) thoracostomy tubes in a large series of cats with pyothorax and to evaluate the efficacy and complications of this technique. As a second objective, we compare the outcome between 2 groups with different management strategies.

## 2. Materials and Methods

Cats with spontaneous pyothorax treated via small-bore thoracostomy tubes between February 2006 and September 2018 were included. Primary surgical care (foreign bodies on initial diagnostic imaging or oesophageal perforation) led to exclusion.

Two groups were compared based on different management strategies.

Cats in the Standard Group (February 2006 to July 2010) initially had a unilateral thoracostomy tube placed and waited for clinical stabilisation over the next few hours to decide if a second thoracostomy tube was needed. A second tube was placed on the contralateral side of the thorax if the cats showed no improvement in their dyspnoea, despite oxygen supplementation and unilateral drainage, after 3–12 h. Cats in the Standard Group received closed-chest lavage with a balanced electrolyte solution. 

Cats in the Intensive Group (August 2010 to September 2018) had bilateral thoracostomy tubes placed as early as possible to completely drain the septic pleural effusion. A second tube was placed if sonography showed residual pleural effusion on the contralateral side of the thorax after the placement of the first tube, allowing for the safe placement of a second tube. Cats in the Intensive Group received closed-chest lavage with a heparinised balanced electrolyte solution. Medical records were reviewed for information regarding signalment, medical history, physical examination findings, diagnostic work-up, treatment, complications and outcomes. Follow-up information was obtained by re-examination or by contacting the owner or referring veterinarian by email or telephone. The diagnosis of pyothorax was based on a purulent to pyogranulomatous pleural exudate with evidence of bacteria in cytology and/or culture. The distribution of the thoracic effusion was assessed by dorsoventral radiography or sonography of the thoracic cavity. Clinical and haematological findings were evaluated with regard to systemic inflammatory response syndrome (SIRS) criteria (Table 1) at presentation; the diagnosis of SIRS was made if ≥3/4 of the markers were positive [10].

Stabilisation included a combination of IV balanced electrolyte solution, supportive oxygen and general supportive care (e.g., analgesia and antiemetics), as needed. All cats received IV broad-spectrum antibiotics. Antibiotic selection was empirical and determined by the clinician in charge of the case.

**Figure 1:**
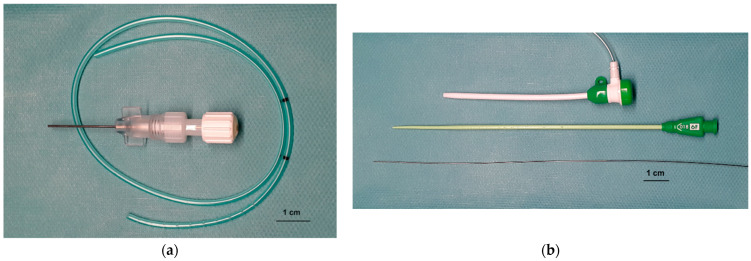
(**a**) and (**b**): Sheath-guided thoracostomy tube system consisting of a small-bore tube and a vascular sheath. (**a**): A 50 cm long 6 F tube with 20 fenestrations at a length of 5 cm. The wall thickness is 0.5 mm and the internal lumen diameter 1 mm. The distance to the fenestration is marked with 2 black markers at 2 cm and 4 cm distance. A Luer lock adapter is included. (**b**) A 6 F valved vascular sheath with its dilator and a 0.018-inch guidewire.

A 6 F sheath-guided thoracostomy tube (Drainage 6 F S00124-7, Walter Veterinaer-Instrumente e. K., Baruth/Mark, Germany; Figure 1a) was inserted in all cats. The site and location of maximum fluid accumulation was determined by ultrasound. Sedation with an opioid (0.2–0.4 mg/kg butorphanol IV/subcutaneous (SC) or 0.005–0.01 mg/kg buprenorphine IV) was induced in all cats. The thoracic wall was clipped and aseptically prepared. Local anaesthesia (0.5 mL lidocaine 2% SC) was administered to all cats. If necessary, additional sedatives were administered at the time of thoracentesis (0.125–0.25 mg/kg midazolam IV and 2.5–5.0 mg/kg ketamine IV). The thorax was punctured with a 20-gauge venous catheter. A thoracentesis was performed via this catheter. Then, a 0.018-inch guidewire was inserted through the catheter. After a stab incision, a 6 F valved vascular sheath with a dilator (Prelude Radial Sheath Introducer 6F PSI-6F-7-018, Merit Medical Systems, Inc., South Jordan, UT, USA; Figure 1b) was inserted into the thorax. The dilator and guidewire were removed and the thoracostomy tube was inserted through the sheath. After removing the sheath, an airtight Luer Lock adapter was attached. The patency of the tube was checked. The tube was fixed with a purse-string suture, followed by a finger trap and a simple interrupted suture about 5 cm apart. Then, the pleural cavity was drained as much as possible via the thoracostomy tube.

The decision for a second tube on the opposite side of the thorax was determined differently in the 2 groups (see group definition above).

A chest dressing and pet cone were applied to protect the tubes from contamination and manipulation by the cat.

After at least 12 h, closed-chest lavage with a warmed (approx. 37 °C) balanced electrolyte solution (10 to 20 mL/kg per cycle and tube) was performed two to four times daily. Pure balanced electrolyte solution (Sterofundin ISO, B. Braun Melsungen AG, Melsungen, Germany) was used in the Standard Group, whereas 10 IU/mL of unfractionated heparin (UFH; Heparin-Natrium Braun 25.000 I.E./5 mL, B. Braun Melsungen AG, Melsungen, Germany) in balanced electrolyte solution was used in the Intensive Group.

In some of the cats in both groups, ampicillin was additionally instilled intrathoracically after lavage (500 mg/chest side q 12 h; Ampicillin-ratiopharm 1.0 g, ratiopharm GmbH, Ulm, Germany).

Further interventions were implemented on a case-by-case basis, such as thoracotomy for severe lung pathology documented on diagnostic imaging or the use of local fibrinolytics (alteplase 0.25 mg/chest side q 24 h; Actilyse 10 mg, Boehringer Ingelheim Pharma GmbH & Co. KG, Biberach an der Riss, Germany or streptokinase 10,000 IU/chest side q 24 h; Streptase 250 000 I.E., CSL Behring GmbH, Marburg, Germany) if lavage was ineffective due to an overly viscous effusion or a lack of improvement.

The thoracostomy tube was electively removed when fluid production had decreased to less than 5 mL/kg/day and there was clinical, radiographic or cytological improvement. The cats were discharged with oral antibiotics for at least 1 month.

Failure of closed-chest lavage was defined as the need for additional therapy by intrathoracic fibrinolysis or thoracotomy.

Tube-related complications (misplacement, blockage or accidental removal) were additionally analysed.

Survival in the early phase (≤48 h), short-term survival (survival to discharge), hospitalisation time (days), long-term survival (survival 1 year after discharge) and recurrence rate were evaluated.

The safety of heparin therapy was assessed by comparing the drop in haematocrit during treatment in both groups.

All statistical analyses were performed using commercially available statistical software (GraphPad Prism, Prism 9 for Windows, Version 9.1.2, San Diego, CA, USA). Data were tested for normal distribution using D’Agostino–Pearson omnibus tests and visual inspections of the data. For descriptive purposes, continuous variables are reported as means ± standard deviations or medians and ranges, depending on the data distribution. Group comparisons were made using *t*-tests, Mann–Whitney tests and Fisher’s exact tests. Values of *p* < 0.05 were considered significant.

## 3. Results

Overall, 45 cats received medical management of their pyothorax with small-bore sheath-guided thoracostomy tubes. Of these, 20 cats were included in the Standard Group, and 25 cats were included in the Intensive Group.

### 3.1. Patient Data and Diagnostic Work-Up

Signalment, medical history and physical examination findings are summarised in Table 2. There were 30 Domestic Shorthairs, 6 Maine Coons, 2 Persians, 2 crossbreeds and 1 British Shorthair, Russian Blue, Balinese, Somali and Kurile Bobtail. Of these, 28 were males (4 intact males), and 17 were females (1 intact female). The median age of the cats was 5 years (range 1 to 18 years).

Main owner concerns were dyspnoea 80% (36/45), lethargy 76% (34/45) and hyporexia 91% (41/45). The median duration of symptoms before presentation was 7 days (range: 1 to 70 days). Of the cats, 82% (37/45) were already pretreated with antibiotics.

The median respiratory rate at the time of presentation was 60 breaths per minute (range: 32 to 124 breaths). The mean heart rate was 171 (±35) beats per minute, and the median body temperature was 38.5 °C (range: 33.3 to 40.8 °C). There were no significant differences between the groups in terms of signalment, medical history or physical examination. All cats were diagnosed radiologically with bilateral effusion.

Complete blood count (CBC), clinical chemistry and pleural fluid analysis, as well as the incidence of certain laboratory abnormalities are shown in Table 3**.** There was no significant difference in the laboratory parameters between the Standard Group and the Intensive Group.

SIRS criteria were available for 96% (43/45) of the cats. SIRS was diagnosed in 40% (17/43) of these cats. In the Standard Group, 50% (9/18) of the cats were diagnosed with SIRS compared to 32% (8/25) in the Intensive Group. This was not a significant difference between the groups (*p* = 0.3443).

Aerobic and anaerobic cultures of the thoracic effusion were performed in 89% (40/45) of the cats. No bacteria could be cultured in 3 cats. All 3 cats were pretreated with antibiotics. In 17 cats, only 1 bacterial species was cultured. In 12 cats, 2 bacterial species were detected; in 6 cats, 3 bacterial species were detected and in 2 cats, 4 bacterial species were detected. The detected bacterial species are listed in Table 4.

### 3.2. Treatment

The following antibiotics were administered: β-lactam antibiotics (amoxicillin/clavulanic acid and ampicillin) in 91% (41/45) of cats; fluoroquinolones (marbofloxacin and enrofloxacin) in 80% (36/45) of cats; metronidazole in 49% (22/45) of cats; amikacin in 4% (2/45) of cats and cefovecin in 4% (2/45) of cats. There was no significant difference between groups in the use of β-lactam antibiotics (*p* > 0.9999) or fluoroquinolones (*p* = 0.1573). Metronidazole was administered significantly more frequently in the Standard Group than in the Intensive Group (16/20 cats and 6/25 cats, respectively; *p* = 0.0003). Ampicillin was administered intrathoracically more often in the Standard Group than in the Intensive Group (18/20 cats and 6/25 cats, respectively; *p* < 0.0001).

### 3.3. Complications and Outcomes

**Figure 2:**
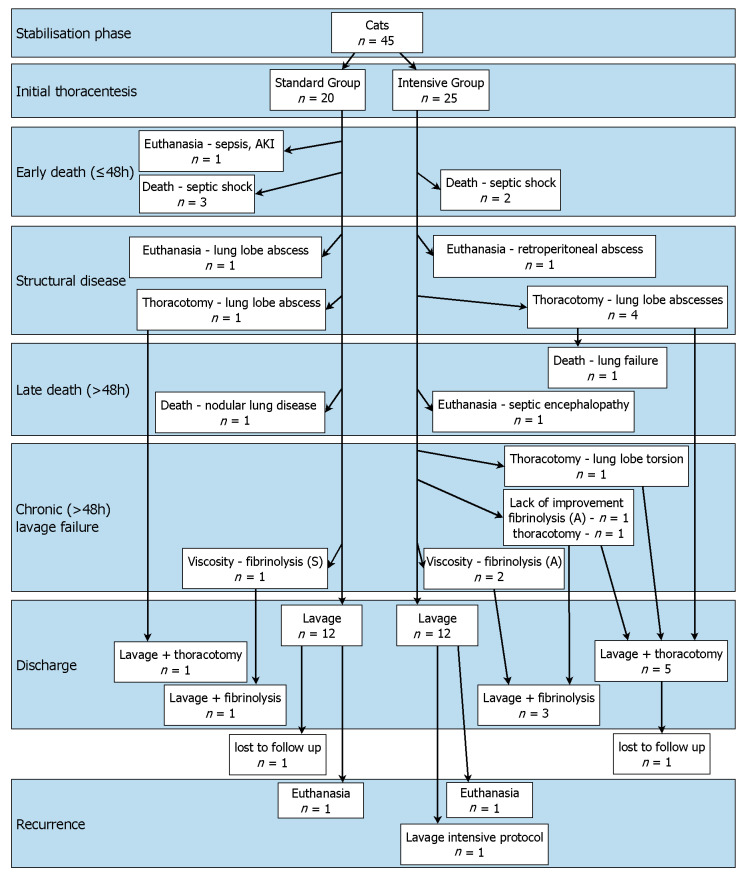
The organigram shows the development of the cats in this study during the hospitalisation. AKI acute kidney injury, S streptokinase, A alteplase.

The population and the development over time are shown in Figure 2. In the early phase (≤48 h), 6 cats died due to the severity of their disease. In the Standard Group, 1 cat was euthanised due to septic shock with a secondary acute kidney injury. Another 3 cats died in this group due to septic shock. In the Intensive Group, 2 cats died due to septic shock. The survival rate in the early phase (≤48 h) was 87% (39/45) in all cats. The survival rate in the Standard Group was 80% (16/20), which was not significantly different (*p* = 0.3830) from the survival rate in the Intensive Group of 92% (23/25).

After the initial stabilisation phase, structural changes requiring surgery were detected by diagnostic imaging in 16% (7/45) of cats. In the Standard Group, 2 cats were diagnosed with lung lobe abscesses. Of these, 1 cat was euthanised (day 6) at the owner’s request, and the other cat was successfully treated by thoracotomy (day 4). In the Intensive Group, 1 cat had a retroperitoneal abscess. This cat was euthanised (day 3) at owner’s request. Another 4 cats in this group required thoracotomies due to lung lobe abscesses (median: day 8, range: 4 to 11 days).

In the course of treatment, 3 more cats died. In the Standard Group, pyothorax of 1 cat initially improved. After removal of the tubes, a severe thoracic effusion (transudate) occurred. At this timepoint, a reticular nodular lung pattern was detected on chest radiographs. This cat died acutely (day 11) and a necropsy was not allowed by the owner. In the Intensive Group, 1 cat was euthanised (day 13) at owner’s request after thoracotomy with lung lobe resection, subtotal pericardiectomy and partial pleurectomy, because the lung function did not improve after 48 h of controlled mechanical ventilation. Histopathological examination revealed chronic purulent pericarditis and pleuritis and multifocal atelectasis with purulent bronchopneumonia. Another cat in the Intensive Group was euthanised (day 5) at the owner’s request because of suspected septic encephalopathy with a brainstem lesion (score 11/18 on the modified Glasgow Coma Scale, assigned to the category with guarded prognosis [11]). Histopathology of the brain was unremarkable.

Forty-three cats survived the first twelve hours after tube placement, so lavage was performed. Failure of closed-chest lavage occurred in 14% (6/43) of the cats. In the Standard Group, 1 cat had a thoracic effusion that was too viscous for adequate drainage, so streptokinase was successfully used intrathoracically (from day 8). In the Intensive Group, lavage failed in 5 cats. Of these, 2 cats had thoracic effusion that was too viscous, so alteplase was successfully used intrathoracically (from day 3 and day 6, respectively). Two cats did not improve sufficiently. In 1 of them, the amount of effusion and the number of cells in the effusion increased during the treatment so that alteplase was successfully instilled intrathoracically (from day 9). The second cat showed persistent bacteria in the effusion and septations on ultrasound examination (day 7). The cat underwent thoracotomy in another clinic. One cat developed lung lobe torsion during closed-chest lavage, so this cat underwent lung lobe resection (day 9).

Overall, 53% (24/45) of the cats were successfully treated with small-bore thoracostomy tubes and closed-chest lavage alone. The 23 cats that were treated according to medical recommendations were discharged after a median of 10 days (range: 7 to 23 days). One cat was discharged early (day 7) at the owner’s request for financial reasons. In the Standard Group, 60% (12/20) of the cats were discharged after a median of 10 days (range: 7 to 23 days). Similarly, in the Intensive Group, 48% (12/25) of the cats were discharged after a median of 10 days (range: 7 to 15 days). The difference in survival to discharge with lavage alone and in length of hospitalisation was not significant between groups (*p* = 0.5503 and *p* = 0.4995, respectively). The hospitalisation of the cat in the Standard Group with closed-chest lavage and thoracotomy lasted 7 days. The cat in the Standard Group with closed-chest lavage and intrathoracic fibrinolysis was hospitalised for 14 days. The hospitalisation of the 5 cats discharged alive from the Intensive Group after closed-chest lavage and thoracotomy lasted a median of 17 days (range: 11 to 20 days). The hospitalisation of the 3 cats in the Intensive Group treated with closed-chest lavage and intrathoracic fibrinolysis lasted 14, 14 and 16 days.

The short-term survival rate (survival to discharge) was 76% (34/45) for all cats. In the Standard Group, 70% (14/20) of the cats survived to discharge and in the Intensive Group 80% (20/25) survived. The difference was not significant (*p* = 0.5001). One cat from the Standard Group was lost to follow up after discharge.

Thirty-three cats had a follow-up for a median of 1222 days (range: 14 to 4645 days).

The recurrence rate was 9% (3/33). Only 8% (1/13) of the cats in the Standard Group showed a relapse (day 14). One cat was euthanised due to financial limitations and concurrent FIV (feline immunodeficiency virus) infection. In the Intensive Group, 10% (2/20) of the discharged cats showed a relapse. The early discharged cat relapsed on day 14 and was euthanised for financial reasons. The second cat relapsed on day 67 and was successfully treated again according to the intensive protocol. This cat had a disease-free follow-up of 1205 days.

One cat was lost to follow-up 295 days after discharge.

After discharge, 94% (30/32) of the cats survived at least 1 year. In the Standard Group, 92% (12/13) of the cats survived more than 1 year and in the Intensive Group 95% (18/19). There was no difference (*p* > 0.9999).

Of the 43 cats with a follow-up, 70% (30/43) survived at least 1 year. In the Standard Group, 63% (12/19) of the cats survived and in the Intensive Group 75% (18/24). There was no significant difference (*p* = 0.5095).

### 3.4. Tube Placement and Complications

In the 45 cats, a total of 59 thoracostomy tubes were placed for initial chest drainage. In the Standard Group, 20 thoracostomy tubes were placed in 20 cats, while in the Intensive Group, 39 thoracostomy tubes were placed in 25 cats. All 59 tubes functioned in terms of drainage of pleural exudate. In 1 cat from the Intensive Group, the perforations of the tubes were partially subcutaneous, so the tube was judged as non-functional for lavage. The tube was removed. The cat was then successfully treated with 1 tube.

During the course, a second tube was placed to empty the thorax on the opposite side in 7 cats in the Standard Group (median: 2 days, range: 1 to 11 days) and in 2 cats in the Intensive Group (day 2 and day 3). In 2 cats of the Intensive Group, a tube had to be replaced. In 1 cat, the tube was blocked (day 6). Another cat was able to pull the tube (day 6) itself. Thus, a total of 67 tubes were in place during the treatment. Of these, 27 tubes were placed in 20 cats of the Standard Group and 40 tubes in 25 cats in the Intensive Group. No complications were observed in the Standard Group. As mentioned above, in the Intensive Group, 1 tube was misplaced (partly subcutaneously), 1 tube was blocked and 1 tube was pulled by the cat. This resulted in a complication rate of 4% (3/67 tubes).

### 3.5. Effect of the Treatment Protocol

Seventeen of the cats received bilateral tubes within 24 h of admission and 82% (14/17) survived. The other 28 cats received only 1 tube (*n* = 23) or the second tube (*n* = 5) was placed after 24 h. Survival to discharge in this group was 71% (20/28). The effect of early bilateral tubes on survival was not significant (*p* = 0.4927).

As mentioned before, lavage could be performed in 43 cats. Lavage with heparinised balanced electrolyte solution was used in 23 cats and 87% (20/23) survived. Lavage with balanced electrolyte solution without UFH was used in 20 cats and 70% (14/20) survived. The effect of different lavage solutions on survival was not significant (*p* = 0.2634)

The haematocrit of the cats decreased with a mean of 0.06 l/l (±0.06) during the treatment. The mean decrease in the Standard Group was 0.09 l/l (±0.06, mean day 6 ± 2, *n* = 11) and in the Intensive Group 0.04 l/l (±0.05, mean day 7 ± 2, *n* = 19). The decrease was significantly lower in the Intensive Group (*p* = 0.0326).

## 4. Discussion

The current study is the first to demonstrate the efficacy and complication rate of small-bore sheath-guided thoracostomy tubes for the treatment of pyothorax in cats. The more intensive protocol with the early goal of bilateral tubes and closed-chest lavage with UFH shows no significant effect.

There is no established standard for the treatment of pyothorax in cats. Parenteral antibiotics are recommended as a starting point, while their administration into the pleural cavity is no longer recommended [12]. In addition, in most studies a closed-chest lavage is performed [1,2,3,4,5,6], which is also recommended in the current guidelines [12].

Different techniques have been described for the placement of thoracostomy tubes for the treatment of pyothorax in cats. In most clinical studies, large-bore tubes (14–16 F) are placed over a rigid trocar under general anaesthesia [1,4,5,6]. Reported complications with these systems include death during general anaesthesia in 10% [4], pneumothorax in 11% [5], lung laceration in 17% [5] and subcutaneous fluid leakage in 6% of cases [5]. An experimental study demonstrates that small-(8 F) and large-bore (16–20 F) tubes are similarly effective in removing fluid or air from the pleural space of canine cadavers [13]. Two studies have been published in cats with pyothorax in which tubes of comparable size (personal communication) were placed over a trocar [3] (10 F) or through a needle [2] (8 F). The trocar technique required general anaesthesia in all cases [3]. The large needle for the 8 F tubes is sharp and may cause injury to the lungs or intrathoracic vessels. In addition, the needle has a larger outer diameter (10 F) than the tube (8 F), which may promote subcutaneous fluid leakage. A wire-guided technique is described for small-bore tubes (6 F) in 2 case series of cats with pyothorax (*n* = 8 [8] and *n* = 10 [9]). These tubes can be placed under sedation using an introducer needle (14 Ga or 18 Ga included in the kit) and a 0.035 inch guidewire [8]. Inadequate drainage effectiveness due to kinking of these tubes is described in 14% of cases (2/14 tubes) [8]. A possible cause could be the thin wall (0.4 mm) in relation to the large inner diameter (1.2 mm) [14].

The sheath-guided tube technique described here is a combination of the advantages of the different tube systems. Anaesthetic complication, lung laceration and fluid leakage did not occur in our cats because the tube system is applicable under local anaesthesia with sedation and because a small (20 Ga) introducer needle, a tiny guidewire (0.018-inch) and a thin-walled sheath are used. Another advantage is the valved sheath, which reduces air entry during placement. Consequently, none of our cats developed a clinically relevant pneumothorax during tube placement, but due to the retrospective nature of our study, small amounts of pneumothorax could not be excluded. In addition, the sheath guidance helps to ensure optimal positioning of the tube. The tube used in this study is constructed for wound drainage with suction, therefore it has a relatively thick wall (0.5 mm). The thick wall (0.5 mm) in relation to the small inner diameter (1 mm) could result in high kink resistance [14]. Nevertheless, due to its multiple fenestration (20 side holes), the tube has a very good drainage performance, which also reduces the risk of blockage. Therefore, medical treatment failure due to tube complications, such as malposition or blockage, occurred in only 1 cat each, and kinking did not happen. One cat removed the tube itself, resulting in a low tube complication rate of 4% (3/67 tubes) in our study. In the literature, such tube complications are described in 11% [4] to 17% [5] of cases with large-bore tubes, 4% [2] to 14% [3] with medium-bore tubes and in 29% [8] with small-bore tubes.

The goal in the Intensive Group was to clear as much as possible of the effusion early in the course of treatment and to improve lavage by using UFH. The reason for using UFH was to prevent the occlusion of thoracostomy tubes by fibrin clots [15]. Closed-chest lavage with UFH has been described in dogs and was associated with a higher long-term (12-month) survival rate [16]. UFH increases fibrinolysis to a small extent, as has been shown in an experimental study in rabbits [17]. It also has an anti-inflammatory effect that prevents the accumulation of leukocytes, as, for example, in ischaemic brain injuries [18]. Heparin is a glycosaminoglycan. A synthetic glycosaminoglycan showed a significant reduction in total cell count, total protein concentration and proinflammatory cytokine concentrations in peritoneal lavage fluid in a model of acute peritoneal inflammation in mice [19].

We observed no difference in the complication rate between the Standard Group and the Intensive Group. The survival rate also did not differ between the treatment groups. This can be explained by several reasons. First, the small number of cats included in the study and the severity of the disease, which leads to a high mortality rate in the first 48 h [6], independent of the different treatment strategies. In addition, communication between both sides of the thorax has been described in cats [20,21], so both sides of the thorax may be adequately drained and lavaged via 1 tube. Single or multiple thoracentesis with parenteral antibiotic treatment has also been described to be curative [1,4].

The overall survival rate for cats treated with sheath-guided tubes is 76%. This is comparable to survival rate in studies using large-bore tubes (14–16 F; 46% to 78%) [1,4,5] or medium-bore tubes (8–10 F; 65 and 72%) [2,3] and to a case series using small-bore tubes (6 F, 88%) [8]. Survival is mainly influenced by 3 factors: death from severe inflammation in the first 48 h and, less frequently at a later stage, structural disease requiring surgical intervention and, finally, tube failure with a need for additional intervention such as thoracotomy or fibrinolysis. Added to this are the financial concerns of the owner, which can lead to losses at all 3 of the aforementioned levels.

In the current study, the acute mortality rate (≤48 h) of 13% is comparable to the rates in other studies of 17% to 22% [1,2]. Pyothorax can lead to sepsis in cats, which is likely the cause of the high mortality rate in the first 48 h [1,22]. In cats, sepsis is defined as meeting SIRS criteria and having a documented source of infection [10,22]. Overall, 40% of cats were SIRS positive with no significant difference between the Standard and Intensive Groups. Future treatment strategies should include more aggressive treatment of this problem.

Another factor is the structural diseases detected by diagnostic imaging. In our cat population, 16% had structural changes, which is more than twice as many cats as in other studies (4% to 6%) [1,2,5]. Surgical intervention for these patients has a good prognosis in all studies, but sometimes owners refuse surgery because of emotional or financial reasons.

Medical treatment fails due to lavage failure in 5% to 9% of cases in studies of cats with pyothorax [1,2,3,4]. In these cases, thoracotomy is usually recommended. In our cat population, lavage failure occurred in 14% of cats. One of these cats required a thoracotomy for lung lobe resection due to lung lobe torsion. Another cat underwent thoracotomy in an external clinic due to lack of improvement. Four other cats received intrathoracic fibrinolysis due to an overly viscous effusion or insufficient improvement. Inflammation leads to an accumulation of extravascular fibrin, as local coagulation is increased while fibrinolysis is decreased. Therefore, intrapleural fibrinolytic therapy has been investigated in humans with empyema [23]. Following this, we have successfully used fibrinolysis at an extrapolated dose in all 4 cats. The use of a higher dose of UFH or earlier use of fibrinolysis may be a treatment option in the future.

A side effect of lavage with heparinised balanced electrolyte solution might have been a bleeding tendency. In both groups, there was a decrease in haematocrit during hospitalisation. Hospital-acquired anaemia is common in cats [24,25]. It is thought that anaemia of inflammatory disease is the most common cause of this finding [26]. The Intensive Group with UHF in the lavage solution had a slightly lower drop in haematocrit, making a bleeding tendency unlikely. A possible reason for the difference could be a small variation in dehydration status between the 2 groups at presentation. For a more objective assessment of the bleeding risk, in the form of an iatrogenic coagulopathy, the measurement of clotting times and/or antifactor Xa activity would be helpful. This should be considered, especially when planning a possible higher UFH dose in the future.

One cat developed lung lobe torsion during lavage. This is rarely described in cats with other types of pleural effusion (especially chylothorax) [27] and should be considered, particularly if the thoracic effusion changes composition.

The relapse rate in our study was 9%, which is similar to that reported in other studies (5% to 8%) [1,2,4,6]. When the analysis included cats that required retreatment, the long-term survival (survival 1 year after discharge) was comparable, with 92% in the Standard Group and 95% in the Intensive Group. Another cat study reported a similar long-term survival rate of 97% [2].

Our investigations have some limitations. First of all, the retrospective character of the study and the large time frame (change in treatment over time) may influence our results. Furthermore, the low statistical power likely affects our results. Although pyothorax is named as the third most common cause of pleural effusion in cats [28,29,30], the condition is rare. This is also reflected in the number of cases in published studies. Despite long observation periods, the number of cases is predominantly less than 40 cats. A prospective study, with case numbers that allow an adequate power of evaluation, is therefore only feasible as a multicentre project. However, in order to plan and justify a meaningful study protocol, an evaluation of our previous data is essential.

## 5. Conclusions

Based on our data, we can show that the therapy of pyothorax with small-bore thoracostomy tubes is comparably successful as the therapy with large- or medium-bore tubes.

In addition, we did not observe an increased complication rate due to bilateral tube placement or closed-chest lavage with a heparinised lavage solution.

Cats treated according to the intensive protocol could be discharged slightly earlier compared to the standard protocol. This group also appeared to have a higher percentage of surviving cats. Larger studies are needed to test the validity of these results.

## Figures and Tables

**Table 1:** Markers and cut-off values for systemic inflammatory response syndrome (SIRS ≥ 3/4 markers).

Marker	Cutoff
Rectal temperature	<37.8 °C or >39.7 °C
Heart rate	<140 beats/minute or >225 beats/minute
Respiratory rate	>40 breaths/minute
WBC count	<5000/μL or >19,500/μL OR >5% band neutrophil fraction

According to Brady et al., 2000 [10].

**Table 2:** Signalment, medical history and physical examination findings.

Parameter	Overall *n* = 45	Standard Group *n* = 20	Intensive Group *n* = 25	*p*-Value
Breed % (*n*)				
Pedigree breed Domestic Shorthair/crossbreed	29 (13/45) 71 (32/45)	20 (4/20) 80 (16/20)	36 (9/25) 64 (16/25)	0.2393
Sex % (*n*)				
Male Female	62 (28/45) 38 (17/45)	45 (9/20) 55 (11/20)	76 (19/25) 24 (6/25)	0.0621
Age ^†^ years (*n*)	5 (1–18) (44)	6 (1–14) (19)	5 (1–18) (25)	0.4539
Main owner concerns % (*n*)				
Hyporexia Dyspnoea Lethargy	91 (41/45) 80 (36/45) 76 (34/45)	95 (19/20) 85 (17/20) 75 (15/20)	88 (22/25) 76 (19/25) 76 (19/25)	0.6174 0.7095 >0.9999
Duration of illness ^†^ days	7 (1–70)	4 (1–70)	7 (1–29)	0.5585
Antimicrobial pretreatment % (*n*)	82 (37/45)	75 (15/20)	88 (22/25)	0.4347
Respiratory rate ^†^ breaths/minute Tachypnoea [>40 breaths/minute] % (*n*)	60 (32–124) 89 (39/44)	52 (36–124) 89 (17/19)	60 (32–100) 88 (22/25)	0.2473 >0.9999
Heart rate * beats/minute Tachycardia [>225 beats/minute] % (*n*) Bradycardia [<140 beats/minute] % (*n*)	171 (±35) 4 (2/45) 16 (7/45)	174 (±40) 10 (2/20) 20 (4/20)	168 (±31) 0 (0/25) 12 (3/25)	0.6095 0.1919 0.6822
Internal body temperature ^†^ °C Fever [>39.7°C] % (*n*) Hypothermia [<37.8°C] % (*n*)	38.5 (33.3–40.8) 14 (6/44) 25 (11/44)	38.7(33.5–40.8) 16 (3/19) 26 (5/19)	38.2(33.3–40.1) 12 (3/25) 24 (6/25)	0.2919 >0.9999 >0.9999

* Data are expressed as means (±standard deviations); ^†^ Data are expressed as medians (ranges).

**Table 3:** Laboratory findings.

Parameter	Overall *n* = 45	Standard Group *n* = 20	Intensive Group *n* = 25	*p*-Value
White blood cell count (G/l) ^†^	19 (2.8–69.5)	21.8 (5.8–69.5)	15.8 (2.8–56.5)	0.3322
Leukocytosis [>19.5 G/l] % (*n*) Leukopenia [<5 G/l] % (*n*)	47 (21/45) 4 (2/45)	55 (11/20) 0 (0/20)	40 (10/25) 8 (2/25)	0.3767 0.4949
Band Neutrophils (G/l) ^†^	3.2 (0–21.6)	3.3 (1.7–19.5)	3.1 (0–21.6)	0.8785
Left shift [>5% band neutrophil fraction] % (*n*)	89 (31/35)	83 (10/12)	91 (21/23)	0.5941
Haematocrit (l/l) *	0.36 (±0.08)	0.39 (±0.07)	0.34 (±0.08)	0.0519
Anaemia [<0.24 l/l] % (*n*)	7 (3/45)	0 (0/20)	12 (3/25)	0.2424
Platelet count (G/l) *	269 (±121)	280 (±128)	261 (±116)	0.6144
Thrombocytosis [>550 G/l] % (*n*) Thrombocytopenia [<180 G/l] % (*n*)	- 27 (12/44) ^#^	- 26 (5/19) ^#^	- 28 (7/25) ^#^	- >0.9999
Urea (mmol/L) ^†^	9.6 (3.8–29.7)	10.2 (3.8–24.8)	8.5 (6.1–29.7)	0.9439
Urea increased [>10.7 mmol/L] % (*n*) Urea decreased [<7.14 mmol/L] % (*n*)	36 (16/44) 23 (10/44)	42 (8/19) 26 (5/19)	32 (8/25) 20 (5/25)	0.5401 0.7234
Creatinine (µmol/L) ^†^	87 (38–402)	87 (48–402)	87 (38–167)	0.7892
Creatinine increased [>168 µmol/L] % (*n*)	5 (2/38)	11 (2/18)	0 (0/20)	0.2176
Calcium, ionized (mmol/L) ^†^	1.2 (0.9–1.8)	1.2 (1.1–1.4)	1.2 (0.9–1.6)	0.9113
Hypercalcaemia [>1.41 mmol/L] % (*n*) Hypocalcaemia [<1.19 mmol] % (*n*)	7 (3/41) 34 (14/41)	0 (0/17) 41 (7/17)	13 (3/24) 29 (7/24)	0.2537 0.5121
Albumin (g/l) *	21 (±4)	21 (±4)	21 (±4)	0.9728
Hypalbuminaemia [<21 g/l] % (*n*)	42 (16/38)	50 (9/18)	35 (7/20)	0.5118
Glucose (mmol/L) ^†^	7 (1.6–19.8)	6.2 (3.5–13.4)	7.4 (1.6–19.8)	0.3962
Hyperglycaemia [>6.11 mmol/L] % (*n*) Hypoglycaemia [<3.89 mmol/L] % (*n*)	63 (27/43) 5 (2/43)	61 (11/18) 6 (1/18)	64 (16/25) 4 (1/25)	>0.9999 >0.9999
Bilirubin (µmol/L) ^†^	5.3 (0.8–61.2)	7.4 (1.2–55.4)	3.6 (0.8–61.2)	0.1494
Hyperbilirubinaemia [>3.4 µmol/L] % (*n*)	63 (24/38)	78 (14/18)	50 (10/20)	0.1008
Lactate (mmol/L) ^†^	3.5 (1.3–10.2)	4.4 (1.8–6.4)	3.3 (1.3–10.2)	0.2762
Hyperlactataemia [>2.2 mmol/L] % (*n*)	85 (23/27)	91 (10/11)	81 (13/16)	0.6239
Pleural fluid cell count (G/l) ^†^ (*n*)	277 (50–607) (34)	286 (55–576) (13)	238 (50–607) (21)	0.6749
Pleural fluid specific gravity ^†^ (*n*)	1034 (1020–1050) (32)	1032 (1024–1048) (13)	1036 (1020–1050) (19)	0.1831
Pleural fluid total solids (g/l) * (*n*)	49.4 (±14.7) (36)	51.8 (±15.5) (16)	47.6 (±14.2) (20)	0.4010

* Data are expressed as means (±standard deviations); ^†^ Data are expressed as medians (ranges); ^#^ all with platelet aggregates.

**Table 4:** Culture results in 40 cats.

Organism	Overall *n* = 40	Standard Group *n* = 17	Intensive Group *n = 23*
Anaerobic	50	20	30
*Actinomyces species*	4	0	4
*Bacteroides species*	7	3	4
*Filifactor villosus*	1	0	1
*Fusobacterium species*	7	3	4
*Lactobacillus species*	1	0	1
*Peptostreptococcus species*	10	6	4
*Porphyromonas species*	2	2	0
*Prevotella species*	7	5	2
*Corynebacterium*	1	0	1
Unidentified anaerobic Gram-positive and Gram-negative cocci	3	0	3
Unidentified anaerobic Gram-positive and Gram-negative rods	7	1	6
Facultative anaerobic	14	8	6
*Pasteurella species*	7	5	2
*Salmonella enterica subsp. group B*	1	1	0
*Staphylococcus species*	4	1	3
*Streptococcus species*	2	1	1
Aerobic	3	2	1
Aerobic bacilli	3	2	1

## Data Availability

The datasets used and/or analysed during the current study are available from the corresponding author upon reasonable request.
